# Late pulmonary embolism in a patient with non-severe COVID-19: case report, value of antithrombotic prophylaxis and literature review

**DOI:** 10.11604/pamj.2021.38.185.28015

**Published:** 2021-02-18

**Authors:** Phany Brunelle Issanga Maloumbi, Ayoub Hassouni, Junior Rocyr Ibara-Onguema, Bouchra Maatof, Franck Bienvenu Ekoba-Othende, Abdelmajid Bouzerda

**Affiliations:** 1 Cardiology Department, The Avicenna Military Hospital, Marrakech, Morocco

**Keywords:** SARS-CoV-2, non-severe COVID-19, late pulmonary embolism, anthithrombotic prophylaxis, case report

## Abstract

Since the onset of the COVID-19 pandemic, several small cohorts have reported the recurrent occurrence of venous thromboembolic disease (VTE), particulary pulmonary embolism, in serious patients hospitalized in intensive care units. We report the case of a patient who presented a minor COVID-19 infection treated on an outpatient basis with good clinical resolution. She developed a pulmonary embolism three weeks after the onset of symptoms. When she was admitted to the emergency room, the two real time reverse transcription polymerase chain reactions (RT-PCRs) performed were negative, moreover the anti-SARS-CoV-2 Immunoglobulin Gs (IgGs) serological test was positive and the chest scanner without and with injection of contrast product showed specific images of COVID-19 with intermediate pulmonary embolism according to the classification of the European society of cardiology (ESC). This observation is interesting since there are not many studies which address the question of the occurrence of late pulmonary embolism in patients with non-severe COVID-19 and raises the discussion on the criteria for the initiation of thromboembolic prophylaxis treatment at the first diagnosis of the disease and duration of that treatment.

## Introduction

COVID-19 is an infectious disease caused by SARS-CoV-2 (severe acute respiratory syndrome coronavirus 2) discovered in December 2019 in Wuhan, China. Following this initial epidemic, the virus spread throughout the world leading to the declaration of a pandemic by the World Health Organization (WHO) on March 11^th^, 2020. Its diagnosis is molecular by polymerase chain reaction (RT-PCR) test on nasopharyngeal swabs [1]. This test, although specific, has less sensitivity, sometimes requiring a chest computed tomography (CT) scan [2]. According to the latest WHO report, COVID-19 has affected more than 93 million people and more than 2 million deaths worldwide and more than 450,000 people in Morocco and more than 7,000 deaths [3].

The clinical spectrum of the disease is very broad, ranging from minor non specific symptoms, such as fever, dry cough and diarrhea, sometimes associated with mild to severe pneumonia with: dyspnea, tachypnea and hypoxia, resulting in approximately 5% of infected patients to severe respiratory distress syndrome (ARDS), shock or extra-pulmonary multiple organ failure including cardiovascular disease [4]. Indeed, an abnormally high incidence of venous thromboembolic events has been observed particularly in patients suffering from severe forms and hospitalized in intensive care [5,6]. But in a minority of patients with non-severe forms of the disease, the silent thrombogenicity associated with SARS-CoV-2 can sometimes complicate the clinical condition of patient despite the recovery [7].

## Patient and observation

**Figure 1:**
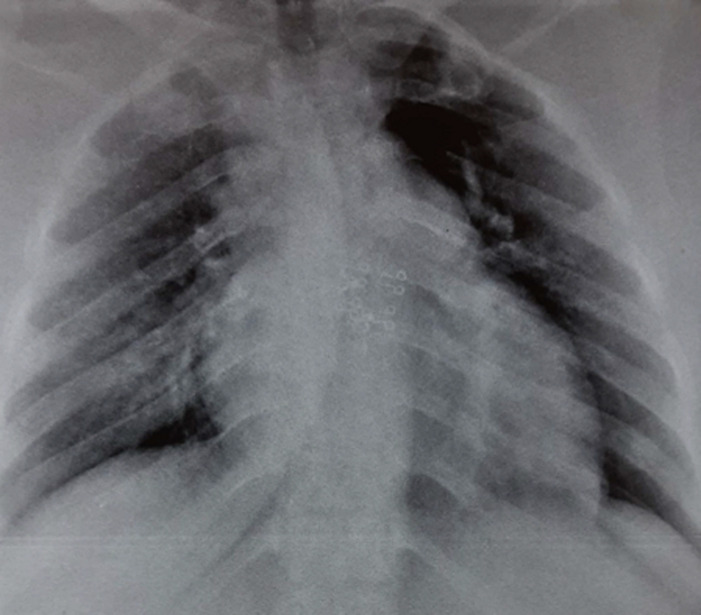
chest X-ray showing right basal pulmonary opacity, cardiomegaly at the expense of the right cavities, convex left middle arch

**Figure 2:**
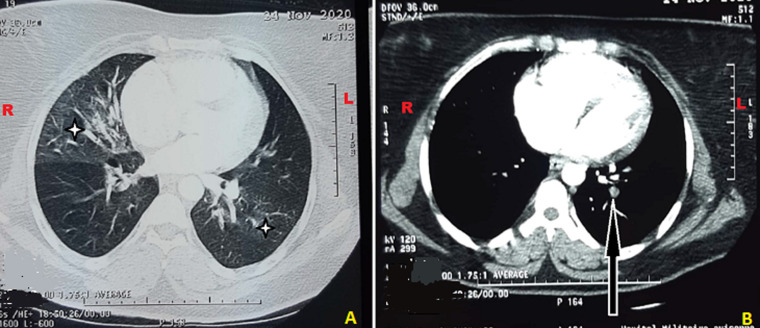
thoracic CT in axial section: A) pulmonary window showing multifocal ground glass foci and scattered of the two pulmonary hemi-fields of subpleural arrangement (white stars); B) with injection of contrast product showing left segmental pulmonary embolism (black arrow)

We report the case of a 38-year-old obese female with a body mass index (BMI) of 35, with no known comorbidity. She reported diarrhea accompanied by fever three weeks before, which revealed a COVID-19 infection, for which she was treated at home without complications. She went to the emergency room on the seventeenth day after the onset of symptoms, with sudden onset dyspnea. The physical examination revealed: Glasgow coma scale 15/15; blood pressure: 127/80mmhg; heart rate: 100 beats/min; respiratory rate: 30 cycles/min; 87% SaO_2_ in ambient air; crackling and wheezing rales at the base of the left lung. Cardiovascular examination was normal, there was no evidence of deep vein thrombosis. Given the epidemiological context, we performed two RT-PCR tests on the first and the second day, all were negative. The anti-SARS-CoV-2 IgG fraction of the serological test was positive. The chest X-ray found left middle arch of the heart convex, right basal pulmonary opacity, bilateral interstitial syndrome (Figure 1). The chest computed tomography with and without injection of contrast product found multifocal ground glass foci (10-25% impairment) of the two pulmonary hemi fields located under pleural with segmental and sub-segmental left pulmonary embolism (Figure 2).

**Figure 3:**
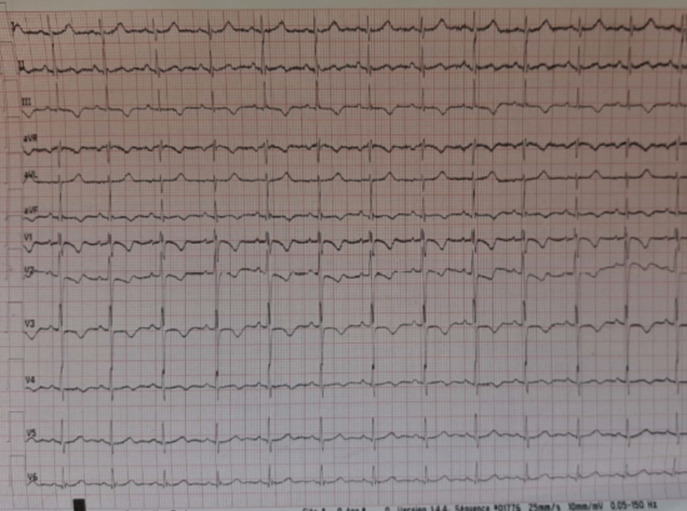
electrocardiogram showing S1Q3 aspect, incomplete right bundle branch block, anteroseptoapical and inferior negative T wave

**Figure 4:**
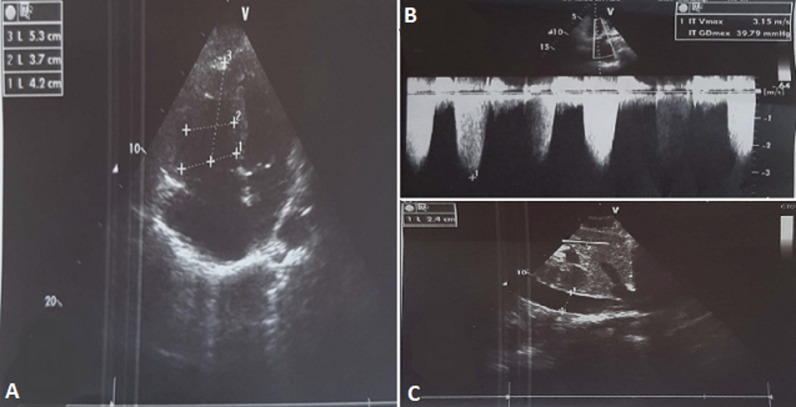
transthoracic ultrasound showing: A) right ventricular dilation; B) tricuspid insufficiency flow; C) dilated lower vena cava

The biological assessment found white blood cells: 13780/UL (N≤10000) with polynuclear neutrophils at 10930/UL (N≤7000); C-reactive protein: 40 (N≤6), troponin: 60ng/L (N≤14), D-dimers: 1000ug/ml (<0.5); creatinine: 9mg/L (6-13), antithrombin, protein S, protein C, factor V Leiden, all were normal. The electrocardiogram found a sinus tachycardia with incomplete right bundle branch block, anteroseptoapical and inferior negative T wave, S1Q3 aspect (Figure 3). The transthoracic ultrasound showed an acute pulmonary heart without right ventricular dysfunction, hypertension at 54 mmhg with dilated lower vena cava (Figure 4). We retained the diagnosis of pulmonary embolism at low intermediate risk on recovered COVID-19 infection. The patient received curative dose of low molecular weight heparin (LMWH) followed by acenocoumarol, oxygen therapy (3L/min). The clinical outcome was favorable after eight days of hospitalization.

## Discussion

Pulmonary embolism is a common complication of COVID-19 infection in severe or hospitalized intensive care unit patients as demonstrated by several studies since the start of the pandemic, which have resulted in the need to put these patients under prolonged prophylactic treatment for up to 45 days depending on the risk of VTE [5,6]. Indeed, a state of hypercoagulability is described during COVID-19 infection, which involves several mechanisms, the most important of which is the release of high plasma levels of pro-inflammatory cytokines at the origin of endothelial dysfunction causing intravascular microthrombosis [8]. This profile of major hypercoagulability encountered during advanced infection with SARS-CoV-2 is quite original. This is more of a coagulopathy than a true disseminated intravascular coagulation as originally described. This notion is supported by the findings of an analysis of hemostasis by thromboelastography (TEG) in COVID-19 patients which noted a state of hypercoagulability associated with major inflammation and a notable elevation of D-dimers. The changes affecting the other parameters (platelet count, prothrombin time, activated partial thromboplastin time, fibrinogen, antithrombin and protein C) being modest [6,9].

Moderate COVID-19 heals after two weeks, according to the WHO. This statement is corroborated by the results of our case which presents two negative RT-PCRs with positive anti-SARS-CoV-2 IgG [10]. However, despite this viral clearance, she developed a pulmonary embolism at the beginning of the third week consistent with the results of Vechi *et al*. [7] in the absence of deep vein thrombosis and no history of VTE.This supports the idea that the duration of the procoagulant effect of COVID-19 after recovery remains fairly undetermined, hence the need to identify possible candidates for “outpatient” thromboprophylaxis based on their clinical profiles. Indeed, obesity is a well-identified risk factor for VTE and arterial disease, which can worsen COVID-19 as can be seen in our patient who has a BMI of 35. Consequently, in the absence of large studies, the Italian society for thrombosis and hemostasis (SISHT), recommends that prophylactic anticoagulation be maintained at home for seven to fourteen days after discharge from hospital or in pre-hospital phase, during home isolation with persistent risk factors such as: BMI>30, history of VTE, active cancer. For this purpose, the use of mid-dose LMWH (i.e. enoxaparin 4000 IU subcutaneous injection every 12 hours) may be considered on an individual basis in patients with multiple risk factors of VTE [11].

However, Vadukul *et al*. reported the occurrence of massive pulmonary embolism in a patient who presented with severe COVID-19 infection a week after discharge from hospital, despite two weeks of antithrombotic prophylaxis during hospitalization [12]. Also, in the series of non-severe COVID-19 cases reported by Vechi *et al*. pulmonary embolism occurs in an average of 19 days [7], which implies that the procoagulant effect of COVID-19 extends over several days see months. Therefore, sticking to a two-week prophylaxis as recommended by SISHT certainly does not solve the problem.

**Informed patient consent:** the patient's oral informed consent was taken.

## Conclusion

Patients with COVID-19 are at increased risk of developing pulmonary embolism (PE), which can occur in both severe and non-severe patients. Although knowledge of the mechanisms of thrombosis formation has increased considerably since the onset of the pandemic, more data on the appropriate anticoagulant therapy for the prevention of thrombosis during COVID-19 infection is awaited in particular from randomized controlled clinical trials, as the recommendations offered so far are based primarily on expert opinion. Extension of thromboprophylaxis after discharge from hospital or during the pre-hospital phase during home self-isolation should be performed according to careful risk/benefit assessment.
